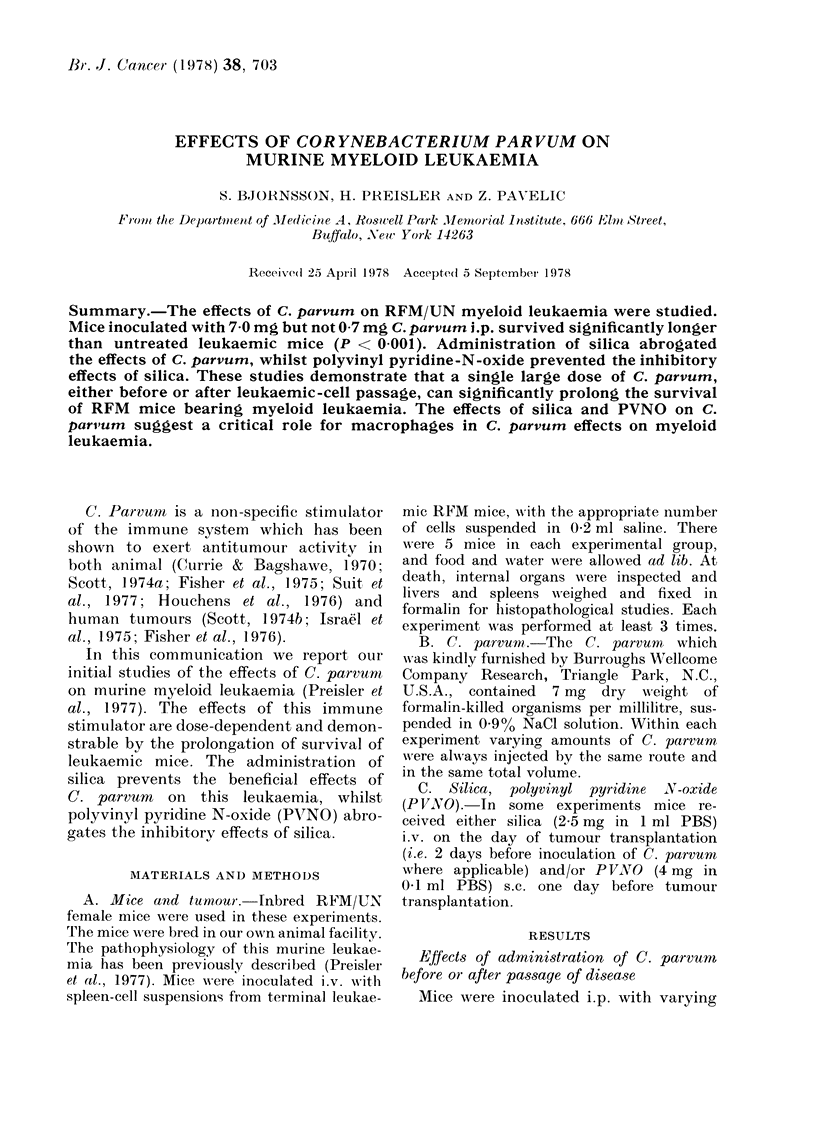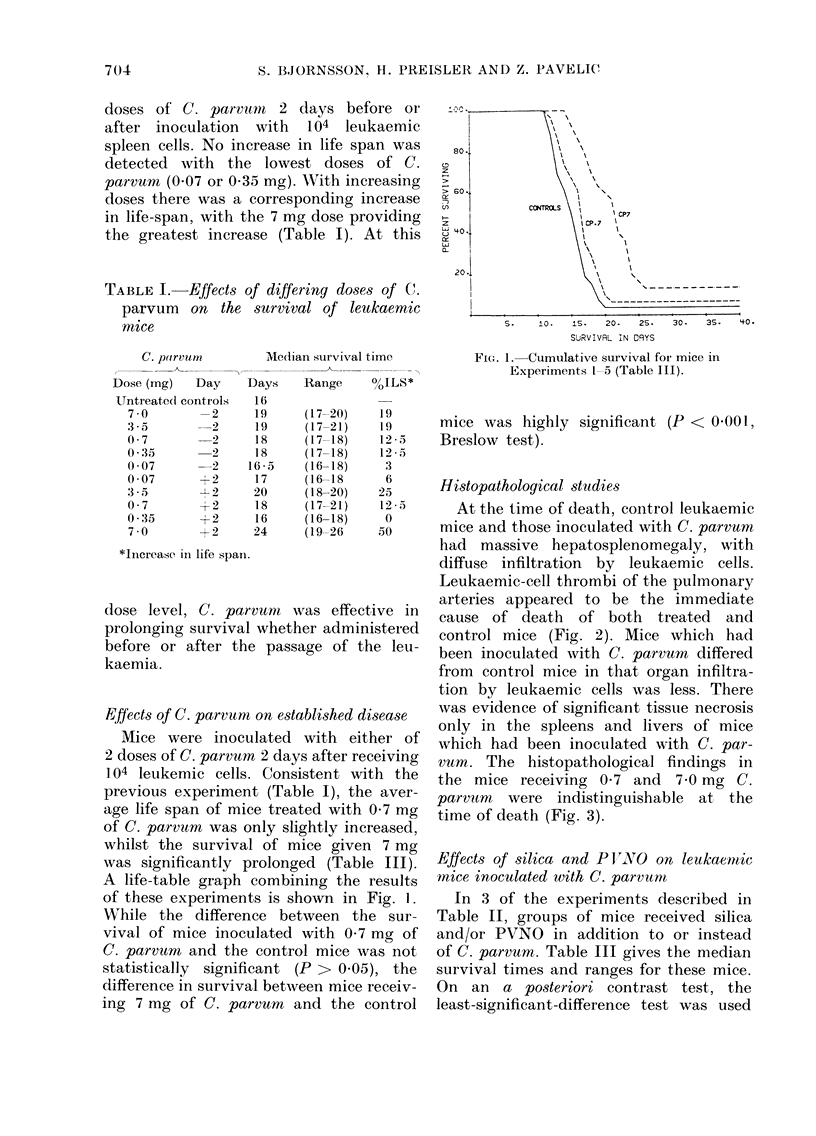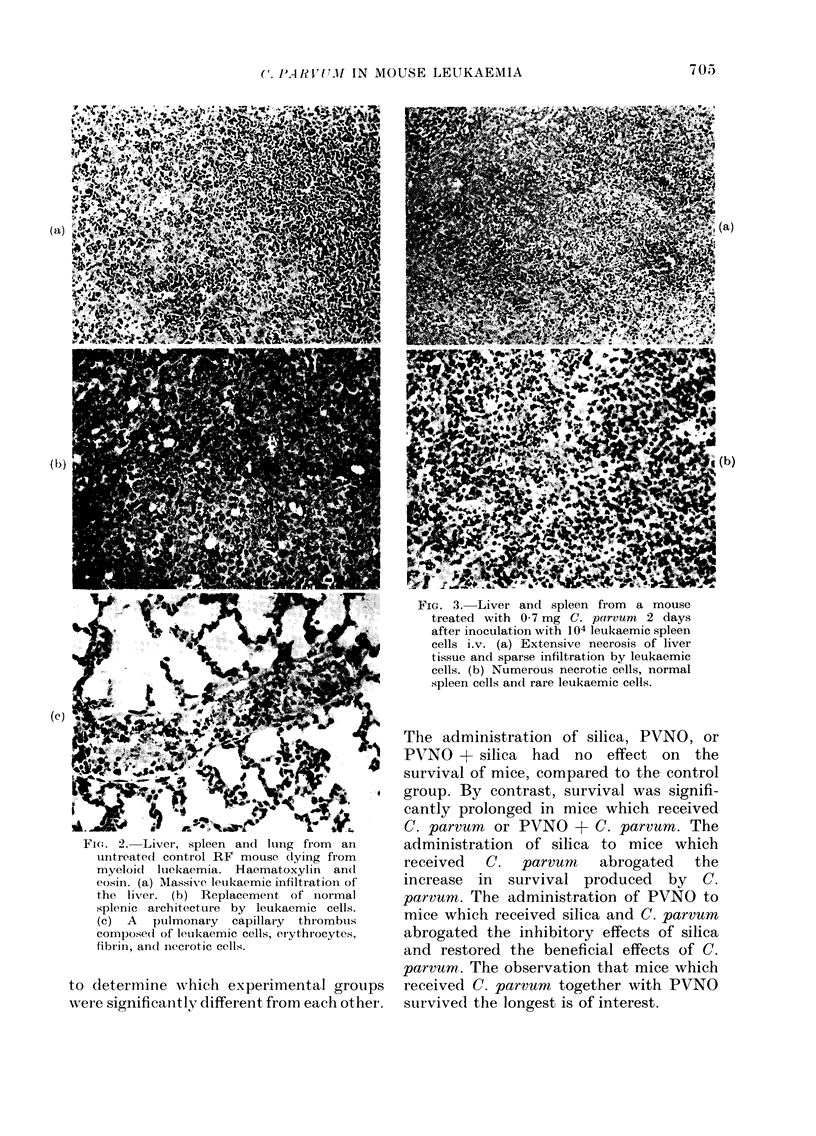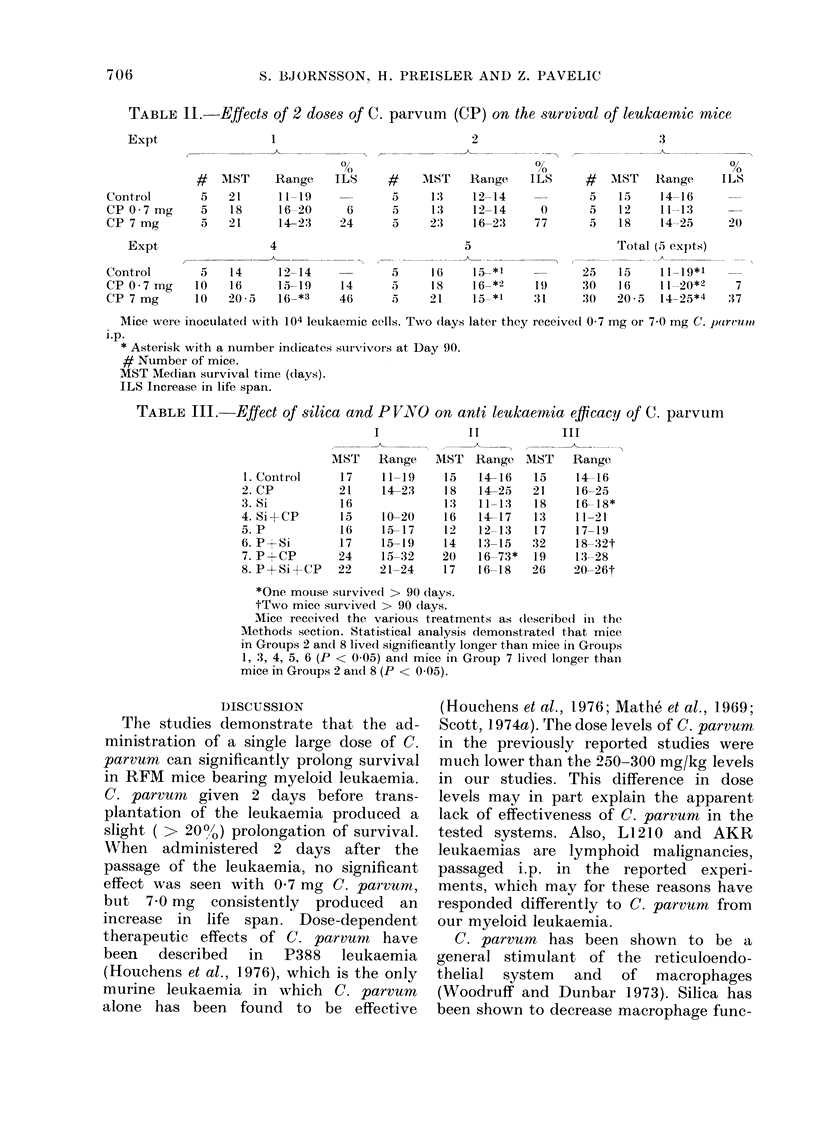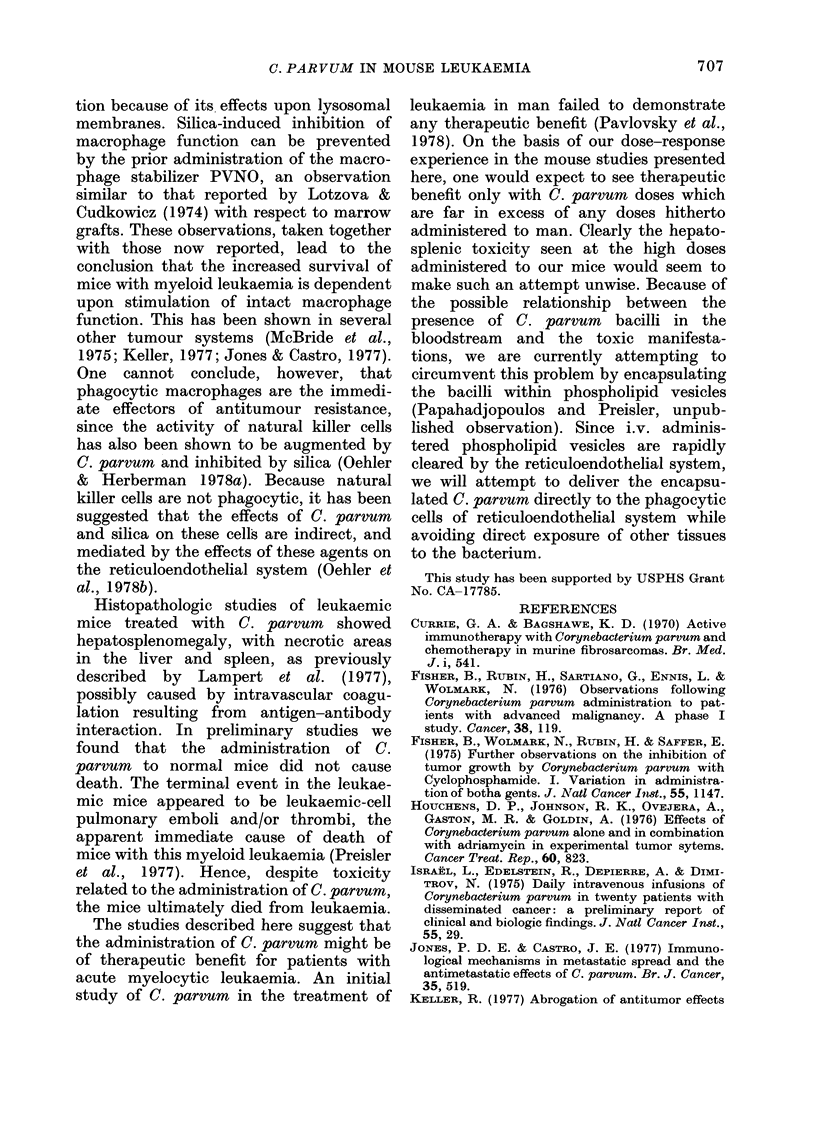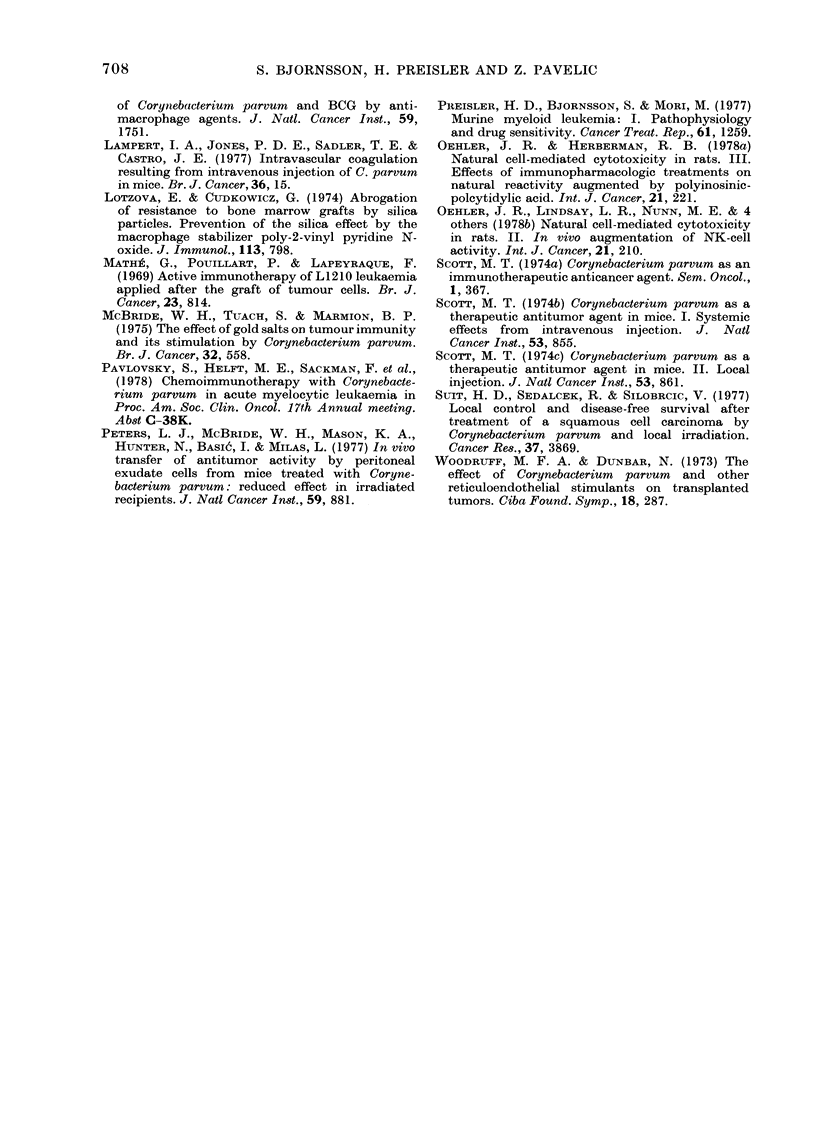# Effects of Corynebacterium parvum on murine myeloid leukaemia.

**DOI:** 10.1038/bjc.1978.276

**Published:** 1978-12

**Authors:** S. Bjornsson, H. Preisler, Z. Pavelic

## Abstract

**Images:**


*Br. J. Cancer* (1978) **38**, 703

### EFFECTS OF *CORYNEBACTERIUM PARVUM* ON MURINE MYELOID LEUKAEMIA

S. BJORNSSON, H. PREISLER AND Z. PAVELIC

*From the Department of Medicine A, Roswell Park Memorial Institute, 666 Elm Street, Buffalo, New York 14263*

Received 25 April 1978 Accepted 5 September 1978

**Summary.—The effects of *C. parvum* on RFM/UN myeloid leukaemia were studied. Mice inoculated with 7·0 mg but not 0·7 mg *C. parvum* i.p. survived significantly longer than untreated leukaemic mice ($P < 0{\cdot}001$). Administration of silica abrogated the effects of *C. parvum*, whilst polyvinyl pyridine-N-oxide prevented the inhibitory effects of silica. These studies demonstrate that a single large dose of *C. parvum*, either before or after leukaemic-cell passage, can significantly prolong the survival of RFM mice bearing myeloid leukaemia. The effects of silica and PVNO on *C. parvum* suggest a critical role for macrophages in *C. parvum* effects on myeloid leukaemia.**

*C. Parvum* is a non-specific stimulator of the immune system which has been shown to exert antitumour activity in both animal (Currie & Bagshawe, 1970; Scott, 1974*a*; Fisher *et al.*, 1975; Suit *et al.*, 1977; Houchens *et al.*, 1976) and human tumours (Scott, 1974*b*; Israël *et al.*, 1975; Fisher *et al.*, 1976).

In this communication we report our initial studies of the effects of *C. parvum* on murine myeloid leukaemia (Preisler *et al.*, 1977). The effects of this immune stimulator are dose-dependent and demonstrable by the prolongation of survival of leukaemic mice. The administration of silica prevents the beneficial effects of *C. parvum* on this leukaemia, whilst polyvinyl pyridine N-oxide (PVNO) abrogates the inhibitory effects of silica.

### MATERIALS AND METHODS

A. *Mice and tumour.*—Inbred RFM/UN female mice were used in these experiments. The mice were bred in our own animal facility. The pathophysiology of this murine leukaemia has been previously described (Preisler *et al.*, 1977). Mice were inoculated i.v. with spleen-cell suspensions from terminal leukaemic RFM mice, with the appropriate number of cells suspended in 0·2 ml saline. There were 5 mice in each experimental group, and food and water were allowed *ad lib.* At death, internal organs were inspected and livers and spleens weighed and fixed in formalin for histopathological studies. Each experiment was performed at least 3 times.

B. *C. parvum.*—The *C. parvum* which was kindly furnished by Burroughs Wellcome Company Research, Triangle Park, N.C., U.S.A., contained 7 mg dry weight of formalin-killed organisms per millilitre, suspended in 0·9% NaCl solution. Within each experiment varying amounts of *C. parvum* were always injected by the same route and in the same total volume.

C. *Silica, polyvinyl pyridine N-oxide (PVNO).*—In some experiments mice received either silica (2·5 mg in 1 ml PBS) i.v. on the day of tumour transplantation (*i.e.* 2 days before inoculation of *C. parvum* where applicable) and/or *PVNO* (4 mg in 0·1 ml PBS) s.c. one day before tumour transplantation.

### RESULTS

### *Effects of administration of C. parvum before or after passage of disease*

Mice were inoculated i.p. with varying doses of *C. parvum* 2 days before or after inoculation with $10^4$ leukaemic spleen cells. No increase in life span was detected with the lowest doses of *C. parvum* (0·07 or 0·35 mg). With increasing doses there was a corresponding increase in life-span, with the 7 mg dose providing the greatest increase (Table I). At this dose level, *C. parvum* was effective in prolonging survival whether administered before or after the passage of the leukaemia.

TABLE I.—*Effects of differing doses of* C. parvum *on the survival of leukaemic mice*

| *C. parvum* | | Median survival time | | |
|---|---|---|---|---|
| Dose (mg) | Day | Days | Range | %ILS* |
| Untreated controls | | 16 | | — |
| 7·0 | −2 | 19 | (17–20) | 19 |
| 3·5 | —2 | 19 | (17–21) | 19 |
| 0·7 | —2 | 18 | (17–18) | 12·5 |
| 0·35 | —2 | 18 | (17–18) | 12·5 |
| 0·07 | —2 | 16·5 | (16–18) | 3 |
| 0·07 | +2 | 17 | (16–18 | 6 |
| 3·5 | +2 | 20 | (18–20) | 25 |
| 0·7 | +2 | 18 | (17–21) | 12·5 |
| 0·35 | +2 | 16 | (16–18) | 0 |
| 7·0 | +2 | 24 | (19–26 | 50 |

*Increase in life span.

*Effects of C. parvum on established disease*

Mice were inoculated with either of 2 doses of *C. parvum* 2 days after receiving $10^4$ leukemic cells. Consistent with the previous experiment (Table I), the average life span of mice treated with 0·7 mg of *C. parvum* was only slightly increased, whilst the survival of mice given 7 mg was significantly prolonged (Table III). A life-table graph combining the results of these experiments is shown in Fig. 1. While the difference between the survival of mice inoculated with 0·7 mg of *C. parvum* and the control mice was not statistically significant ($P > 0 \cdot 05$), the difference in survival between mice receiving 7 mg of *C. parvum* and the control mice was highly significant ($P < 0 \cdot 001$, Breslow test).

FIG. 1.—Cumulative survival for mice in Experiments 1–5 (Table III).

*Histopathological studies*

At the time of death, control leukaemic mice and those inoculated with *C. parvum* had massive hepatosplenomegaly, with diffuse infiltration by leukaemic cells. Leukaemic-cell thrombi of the pulmonary arteries appeared to be the immediate cause of death of both treated and control mice (Fig. 2). Mice which had been inoculated with *C. parvum* differed from control mice in that organ infiltration by leukaemic cells was less. There was evidence of significant tissue necrosis only in the spleens and livers of mice which had been inoculated with *C. parvum*. The histopathological findings in the mice receiving 0·7 and 7·0 mg *C. parvum* were indistinguishable at the time of death (Fig. 3).

*Effects of silica and PVNO on leukaemic mice inoculated with C. parvum*

In 3 of the experiments described in Table II, groups of mice received silica and/or PVNO in addition to or instead of *C. parvum*. Table III gives the median survival times and ranges for these mice. On an *a posteriori* contrast test, the least-significant-difference test was used

FIG. 2.—Liver, spleen and lung from an untreated control RF mouse dying from myeloid luekaemia. Haematoxylin and eosin. (a) Massive leukaemic infiltration of the liver. (b) Replacement of normal splenic architecture by leukaemic cells. (c) A pulmonary capillary thrombus composed of leukaemic cells, erythrocytes, fibrin, and necrotic cells.

to determine which experimental groups were significantly different from each other.

FIG. 3.—Liver and spleen from a mouse treated with 0·7 mg *C. parvum* 2 days after inoculation with $10^4$ leukaemic spleen cells i.v. (a) Extensive necrosis of liver tissue and sparse infiltration by leukaemic cells. (b) Numerous necrotic cells, normal spleen cells and rare leukaemic cells.

The administration of silica, PVNO, or PVNO + silica had no effect on the survival of mice, compared to the control group. By contrast, survival was significantly prolonged in mice which received *C. parvum* or PVNO + *C. parvum*. The administration of silica to mice which received *C. parvum* abrogated the increase in survival produced by *C. parvum*. The administration of PVNO to mice which received silica and *C. parvum* abrogated the inhibitory effects of silica and restored the beneficial effects of *C. parvum*. The observation that mice which received *C. parvum* together with PVNO survived the longest is of interest.

TABLE II.—*Effects of 2 doses of* C. parvum (CP) *on the survival of leukaemic mice*

| Expt | 1 | | | | 2 | | | | 3 | | | |
|---|---|---|---|---|---|---|---|---|---|---|---|---|
| | # | MST | Range | % ILS | # | MST | Range | % ILS | # | MST | Range | % ILS |
| Control | 5 | 21 | 11–19 | — | 5 | 13 | 12–14 | — | 5 | 15 | 14–16 | — |
| CP 0·7 mg | 5 | 18 | 16–20 | 6 | 5 | 13 | 12–14 | 0 | 5 | 12 | 11–13 | — |
| CP 7 mg | 5 | 21 | 14–23 | 24 | 5 | 23 | 16–23 | 77 | 5 | 18 | 14–25 | 20 |

| Expt | 4 | | | | 5 | | | | Total (5 expts) | | | |
|---|---|---|---|---|---|---|---|---|---|---|---|---|
| Control | 5 | 14 | 12–14 | — | 5 | 16 | 15–*1 | — | 25 | 15 | 11–19*1 | — |
| CP 0·7 mg | 10 | 16 | 15–19 | 14 | 5 | 18 | 16–*2 | 19 | 30 | 16 | 11–20*2 | 7 |
| CP 7 mg | 10 | 20·5 | 16–*3 | 46 | 5 | 21 | 15–*1 | 31 | 30 | 20·5 | 14–25*4 | 37 |

Mice were inoculated with $10^4$ leukaemic cells. Two days later they received 0·7 mg or 7·0 mg *C. parvum* i.p.
* Asterisk with a number indicates survivors at Day 90.
### Number of mice.
MST Median survival time (days).
ILS Increase in life span.

TABLE III.—*Effect of silica and PVNO on anti leukaemia efficacy of* C. parvum

| | I | | II | | III | |
|---|---|---|---|---|---|---|
| | MST | Range | MST | Range | MST | Range |
| 1. Control | 17 | 11–19 | 15 | 14–16 | 15 | 14–16 |
| 2. CP | 21 | 14–23 | 18 | 14–25 | 21 | 16–25 |
| 3. Si | 16 | | 13 | 11–13 | 18 | 16–18* |
| 4. Si+CP | 15 | 10–20 | 16 | 14–17 | 13 | 11–21 |
| 5. P | 16 | 15–17 | 12 | 12–13 | 17 | 17–19 |
| 6. P+Si | 17 | 15–19 | 14 | 13–15 | 32 | 18–32† |
| 7. P+CP | 24 | 15–32 | 20 | 16–73* | 19 | 13–28 |
| 8. P+Si+CP | 22 | 21–24 | 17 | 16–18 | 26 | 20–26† |

*One mouse survived > 90 days.
†Two mice survived > 90 days.
Mice received the various treatments as described in the Methods section. Statistical analysis demonstrated that mice in Groups 2 and 8 lived significantly longer than mice in Groups 1, 3, 4, 5, 6 ($P < 0·05$) and mice in Group 7 lived longer than mice in Groups 2 and 8 ($P < 0·05$).

### DISCUSSION

The studies demonstrate that the administration of a single large dose of *C. parvum* can significantly prolong survival in RFM mice bearing myeloid leukaemia. *C. parvum* given 2 days before transplantation of the leukaemia produced a slight ( > 20%) prolongation of survival. When administered 2 days after the passage of the leukaemia, no significant effect was seen with 0·7 mg *C. parvum*, but 7·0 mg consistently produced an increase in life span. Dose-dependent therapeutic effects of *C. parvum* have been described in P388 leukaemia (Houchens *et al.*, 1976), which is the only murine leukaemia in which *C. parvum* alone has been found to be effective (Houchens *et al.*, 1976; Mathé *et al.*, 1969; Scott, 1974*a*). The dose levels of *C. parvum* in the previously reported studies were much lower than the 250–300 mg/kg levels in our studies. This difference in dose levels may in part explain the apparent lack of effectiveness of *C. parvum* in the tested systems. Also, L1210 and AKR leukaemias are lymphoid malignancies, passaged i.p. in the reported experiments, which may for these reasons have responded differently to *C. parvum* from our myeloid leukaemia.

*C. parvum* has been shown to be a general stimulant of the reticuloendothelial system and of macrophages (Woodruff and Dunbar 1973). Silica has been shown to decrease macrophage function because of its effects upon lysosomal membranes. Silica-induced inhibition of macrophage function can be prevented by the prior administration of the macrophage stabilizer PVNO, an observation similar to that reported by Lotzova & Cudkowicz (1974) with respect to marrow grafts. These observations, taken together with those now reported, lead to the conclusion that the increased survival of mice with myeloid leukaemia is dependent upon stimulation of intact macrophage function. This has been shown in several other tumour systems (McBride *et al.*, 1975; Keller, 1977; Jones & Castro, 1977). One cannot conclude, however, that phagocytic macrophages are the immediate effectors of antitumour resistance, since the activity of natural killer cells has also been shown to be augmented by *C. parvum* and inhibited by silica (Oehler & Herberman 1978*a*). Because natural killer cells are not phagocytic, it has been suggested that the effects of *C. parvum* and silica on these cells are indirect, and mediated by the effects of these agents on the reticuloendothelial system (Oehler *et al.*, 1978*b*).

Histopathologic studies of leukaemic mice treated with *C. parvum* showed hepatosplenomegaly, with necrotic areas in the liver and spleen, as previously described by Lampert *et al.* (1977), possibly caused by intravascular coagulation resulting from antigen–antibody interaction. In preliminary studies we found that the administration of *C. parvum* to normal mice did not cause death. The terminal event in the leukaemic mice appeared to be leukaemic-cell pulmonary emboli and/or thrombi, the apparent immediate cause of death of mice with this myeloid leukaemia (Preisler *et al.*, 1977). Hence, despite toxicity related to the administration of *C. parvum*, the mice ultimately died from leukaemia.

The studies described here suggest that the administration of *C. parvum* might be of therapeutic benefit for patients with acute myelocytic leukaemia. An initial study of *C. parvum* in the treatment of leukaemia in man failed to demonstrate any therapeutic benefit (Pavlovsky *et al.*, 1978). On the basis of our dose–response experience in the mouse studies presented here, one would expect to see therapeutic benefit only with *C. parvum* doses which are far in excess of any doses hitherto administered to man. Clearly the hepatosplenic toxicity seen at the high doses administered to our mice would seem to make such an attempt unwise. Because of the possible relationship between the presence of *C. parvum* bacilli in the bloodstream and the toxic manifestations, we are currently attempting to circumvent this problem by encapsulating the bacilli within phospholipid vesicles (Papahadjopoulos and Preisler, unpublished observation). Since i.v. administered phospholipid vesicles are rapidly cleared by the reticuloendothelial system, we will attempt to deliver the encapsulated *C. parvum* directly to the phagocytic cells of reticuloendothelial system while avoiding direct exposure of other tissues to the bacterium.

This study has been supported by USPHS Grant No. CA–17785.

### REFERENCES

CURRIE, G. A. & BAGSHAWE, K. D. (1970) Active immunotherapy with *Corynebacterium parvum* and chemotherapy in murine fibrosarcomas. *Br. Med. J.* i, 541.

FISHER, B., RUBIN, H., SARTIANO, G., ENNIS, L. & WOLMARK, N. (1976) Observations following *Corynebacterium parvum* administration to patients with advanced malignancy. A phase I study. *Cancer*, **38**, 119.

FISHER, B., WOLMARK, N., RUBIN, H. & SAFFER, E. (1975) Further observations on the inhibition of tumor growth by *Corynebacterium parvum* with Cyclophosphamide. I. Variation in administration of botha gents. *J. Natl Cancer Inst.*, **55**, 1147.

HOUCHENS, D. P., JOHNSON, R. K., OVEJERA, A., GASTON, M. R. & GOLDIN, A. (1976) Effects of *Corynebacterium parvum* alone and in combination with adriamycin in experimental tumor sytems. *Cancer Treat. Rep.*, **60**, 823.

ISRAËL, L., EDELSTEIN, R., DEPIERRE, A. & DIMITROV, N. (1975) Daily intravenous infusions of *Corynebacterium parvum* in twenty patients with disseminated cancer: a preliminary report of clinical and biologic findings. *J. Natl Cancer Inst.*, **55**, 29.

JONES, P. D. E. & CASTRO, J. E. (1977) Immunological mechanisms in metastatic spread and the antimetastatic effects of *C. parvum*. *Br. J. Cancer*, **35**, 519.

KELLER, R. (1977) Abrogation of antitumor effects of *Corynebacterium parvum* and BCG by anti-macrophage agents. *J. Natl. Cancer Inst.*, **59**, 1751.

LAMPERT, I. A., JONES, P. D. E., SADLER, T. E. & CASTRO, J. E. (1977) Intravascular coagulation resulting from intravenous injection of *C. parvum* in mice. *Br. J. Cancer*, **36**, 15.

LOTZOVA, E. & CUDKOWICZ, G. (1974) Abrogation of resistance to bone marrow grafts by silica particles. Prevention of the silica effect by the macrophage stabilizer poly-2-vinyl pyridine N-oxide. *J. Immunol.*, **113**, 798.

MATHÉ, G., POUILLART, P. & LAPEYRAQUE, F. (1969) Active immunotherapy of L1210 leukaemia applied after the graft of tumour cells. *Br. J. Cancer*, **23**, 814.

MCBRIDE, W. H., TUACH, S. & MARMION, B. P. (1975) The effect of gold salts on tumour immunity and its stimulation by *Corynebacterium parvum*. *Br. J. Cancer*, **32**, 558.

PAVLOVSKY, S., HELFT, M. E., SACKMAN, F. *et al.*, (1978) Chemoimmunotherapy with *Corynebacterium parvum* in acute myelocytic leukaemia in *Proc. Am. Soc. Clin. Oncol. 17th Annual meeting. Abst* **C-38K.**

PETERS, L. J., MCBRIDE, W. H., MASON, K. A., HUNTER, N., BASIĆ, I. & MILAS, L. (1977) *In vivo* transfer of antitumor activity by peritoneal exudate cells from mice treated with *Corynebacterium parvum:* reduced effect in irradiated recipients. *J. Natl Cancer Inst.*, **59**, 881.

PREISLER, H. D., BJORNSSON, S. & MORI, M. (1977) Murine myeloid leukemia: I. Pathophysiology and drug sensitivity. *Cancer Treat. Rep.*, **61**, 1259.

OEHLER, J. R. & HERBERMAN, R. B. (1978*a*) Natural cell-mediated cytotoxicity in rats. III. Effects of immunopharmacologic treatments on natural reactivity augmented by polyinosinic-polcytidylic acid. *Int. J. Cancer*, **21**, 221.

OEHLER, J. R., LINDSAY, L. R., NUNN, M. E. & 4 others (1978*b*) Natural cell-mediated cytotoxicity in rats. II. *In vivo* augmentation of NK-cell activity. *Int. J. Cancer*, **21**, 210.

SCOTT, M. T. (1974*a*) *Corynebacterium parvum* as an immunotherapeutic anticancer agent. *Sem. Oncol.*, **1**, 367.

SCOTT, M. T. (1974*b*) *Corynebacterium parvum* as a therapeutic antitumor agent in mice. I. Systemic effects from intravenous injection. *J. Natl Cancer Inst.*, **53**, 855.

SCOTT, M. T. (1974*c*) *Corynebacterium parvum* as a therapeutic antitumor agent in mice. II. Local injection. *J. Natl Cancer Inst.*, **53**, 861.

SUIT, H. D., SEDALCEK, R. & SILOBRCIC, V. (1977) Local control and disease-free survival after treatment of a squamous cell carcinoma by *Corynebacterium parvum* and local irradiation. *Cancer Res.*, **37**, 3869.

WOODRUFF, M. F. A. & DUNBAR, N. (1973) The effect of *Corynebacterium parvum* and other reticuloendothelial stimulants on transplanted tumors. *Ciba Found. Symp.*, **18**, 287.